# Using pre-screening methods for an effective and reliable site characterization at megasites

**DOI:** 10.1007/s11356-015-4649-6

**Published:** 2015-05-17

**Authors:** Mette Algreen, Mariusz Kalisz, Marcel Stalder, Eugeniu Martac, Janusz Krupanek, Stefan Trapp, Stephan Bartke

**Affiliations:** Department of Environmental Engineering, Technical University of Denmark, 2800 Kgs., Lyngby, Denmark; Institute for Ecology of Industrial Areas, 6 Kossutha Street, Katowice, Poland; SolGeo AG, Dornacherplatz 3, 4501 Solothurn, Switzerland; Fugro Consult GmbH, Volkmaroder Str. 8c, 38104 Braunschweig, Germany; Helmholtz Centre for Environmental Research—UFZ, Permoserstr. 15, 04318 Leipzig, Germany

**Keywords:** Contamination, Tree core, Probe technologies, Brownfields, Phytoscreening, Direct-push, Soil gas, Site characterization

## Abstract

This paper illustrates the usefulness of pre-screening methods for an effective characterization of polluted sites. We applied a sequence of site characterization methods to a former Soviet military airbase with likely fuel and benzene, toluene, ethylbenzene, and xylene (BTEX) contamination in shallow groundwater and subsoil. The methods were (i) phytoscreening with tree cores; (ii) soil gas measurements for CH_4_, O_2_, and photoionization detector (PID); (iii) direct-push with membrane interface probe (MIP) and laser-induced fluorescence (LIF) sensors; (iv) direct-push sampling; and (v) sampling from soil and from groundwater monitoring wells. Phytoscreening and soil gas measurements are rapid and inexpensive pre-screening methods. Both indicated subsurface pollution and hot spots successfully. The direct-push sensors yielded 3D information about the extension and the volume of the subsurface plume. This study also expanded the applicability of tree coring to BTEX compounds and tested the use of high-resolution direct-push sensors for light hydrocarbons. Comparison of screening results to results from conventional soil and groundwater sampling yielded in most cases high rank correlation and confirmed the findings. The large-scale application of non- or low-invasive pre-screening can be of help in directing and focusing the subsequent, more expensive investigation methods. The rapid pre-screening methods also yielded useful information about potential remediation methods. Overall, we see several benefits of a stepwise screening and site characterization scheme, which we propose in conclusion.

**Electronic supplementary material:**

The online version of this article (doi:10.1007/s11356-015-4649-6) contains supplementary material, which is available to authorized users.

## Introduction

Megasites are per definition especially large and prominent brownfields, typically with several pollution sources with various contaminants (Schädler et al. 2012; Schirmer et al. 2012). Their sustainable regeneration demands to carefully consider the local complexities and uncertainties (Bartke and Schwarze 2015). Investors shy away from regeneration which involves the removal of actual or potential pollutions originating from previous use, because these can seriously impair the marketability of contaminated land. The reduced merchantability does not depend so much on the (level of) expected remediation costs but rather on their uncertainty and the remaining effect of stigmatization—an effect that can be reduced by improved site characterization (Bartke 2011). Conventional site characterization approaches are based on sampling soil and groundwater from bore holes and monitoring wells. This tends to be time consuming and costly. At the same time, these approaches may involve uncertainties due to insufficient historical data or sampling density owed to limited budgets. The subsequent risk assessment may then be inaccurate and the results doubtful. Uncertainties can be reduced by applying a denser sampling grid, which, however, may be very expensive when applying traditional methods for large plots such as megasites. If contaminated properties are to be merchantable and reactivated, economically efficient site characterization strategies are a prerequisite.

Every site is unique with respect to the contaminants and their behavior under the conditions specific to each site. Therefore, the methods to characterize and monitor a site need to be tailored to the site-specific conditions (French et al. 2014). Several rapid, low- or non-invasive and cost-efficient techniques have been developed recently and can now be applied as part of the screening and monitoring strategy for megasites (Rein et al. 2011; Rein et al. 2015, submitted; Kästner et al. 2012). Each screening method is related to a different level of precision and delivers different information about the contamination status and also on the ongoing processes at the site. We therefore assessed the opportunities of an optimized site characterization using the information gathered from fast and non-expensive pre-screening methods. In this study, the pre-screening methods of tree coring, soil gas measuring, and direct-push (DP) with high-resolution technologies, membrane interface probe (MIP), and laser-induced fluorescence (LIF) sensors have been applied on a former Soviet military airbase near the city of Szprotawa in southwestern Poland.

*Phytoscreening by tree coring* is a qualitative and semi-quantitative method using trees as bioindicators for subsurface pollution. The technique takes advantage of the uptake and translocation of water from soil and groundwater by trees, and of herein dissolved pollutants. By sampling and analyzing a core from the stem, subsurface pollution can be detected. So far, this pre-screening method has mainly been applied at sites contaminated with chlorinated solvents (Wittlingerova et al. 2013; Sorek et al. 2008; Larsen et al. 2008; Gopalakrishnan et al. 2007; Ma and Burken 2002; Vroblesky et al. 1999). For a couple of years, the feasibility of this method to detect other compounds such as heavy metals and benzene, toluene, ethylbenzene, and xylenes (BTEX) has been of scientific interest (Algreen et al. 2012, 2014; Wilson et al. 2013; Sorek et al. 2008). Results have not always been convincing, and more research on the feasibility of the method is needed. Tree coring requires a minimum of sampling equipment. It is particularly well suited for forested areas and can also be applied in inaccessible, swampy, or remote areas as long as there are trees. It is a non-invasive technique, which is of advantage if there are cables, pipes, or explosives in the underground (Algreen and Trapp 2014). Also, the lack of trees, their deformation, or miserable growth can show high levels of toxic substances in the underground (Trapp et al. 2001).

*Soil gas measurement* is a rapid semi-quantitative method restricted to volatile contaminants in the vadose zone. During sampling, the gas contained in the interstitial spaces of the soil is extracted from a temporary or permanent probe and analyzed on site or in the laboratory. Soil gas measurements are offered commercially for a variety of volatile organic compounds (VOCs) including chlorinated hydrocarbons (e.g., Bishop et al. 1990; Rivett 1995) and petroleum derivatives like BTEX (Caldwell et al. 2012; Ramalho et al. 2014). The method allows real-time on-site measurements, which facilitate a higher degree of flexibility in the field. Besides BTEX and VOC, also methane, CO_2_, and oxygen levels can be monitored. High methane and low oxygen levels originate from aerobic biodegradation processes.

*Direct-push*-based technologies were developed for a variety of drilling methods with pushing or hammering options to enable both screening and in-detail subsurface investigations in comparatively short time periods and at relatively low costs. This technique is performed by pushing and hammering small-diameter hollow steel rods into the ground to acquire high-resolution depth profiles of different parameters. Direct-push high-resolution technologies are used commercially both for site screening and for detailed subsurface investigations. The application of membrane interface probe (MIP) and laser-induced fluorescence (LIF) sensors has strongly increased during recent years (Dietrich and Leven 2006; Jacobs et al. 2000; Pitkin et al. 1999; ASTM 1998, U.S. EPA 1998). The DP method also yields information about the vertical distribution of subsurface contaminations.

*The purpose of this study* has been to investigate the potential of the different pre-screening methods to obtain contamination levels at megasites, and to yield information on remediation options. The application of multiple pre-screening methods allows to sample in a denser grid and thus to obtain more data. This will minimize the risk of overlooking hot spots. Moreover, subsequent cost-intense methods like soil and groundwater sampling can be targeted to the most relevant areas. This makes site characterization more efficient and reliable, despite the fact that even less effort is required. Finally, the results obtained by the various pre-screening methods were compared statistically with each other as well as with the results of the conventional soil and groundwater sampling. The conclusions on remediation options that can be drawn from the outcome of pre-screening will be reported in a separate publication (Clausen et al. 2015).

## Materials and methods

### Site description

**Fig. 1:**
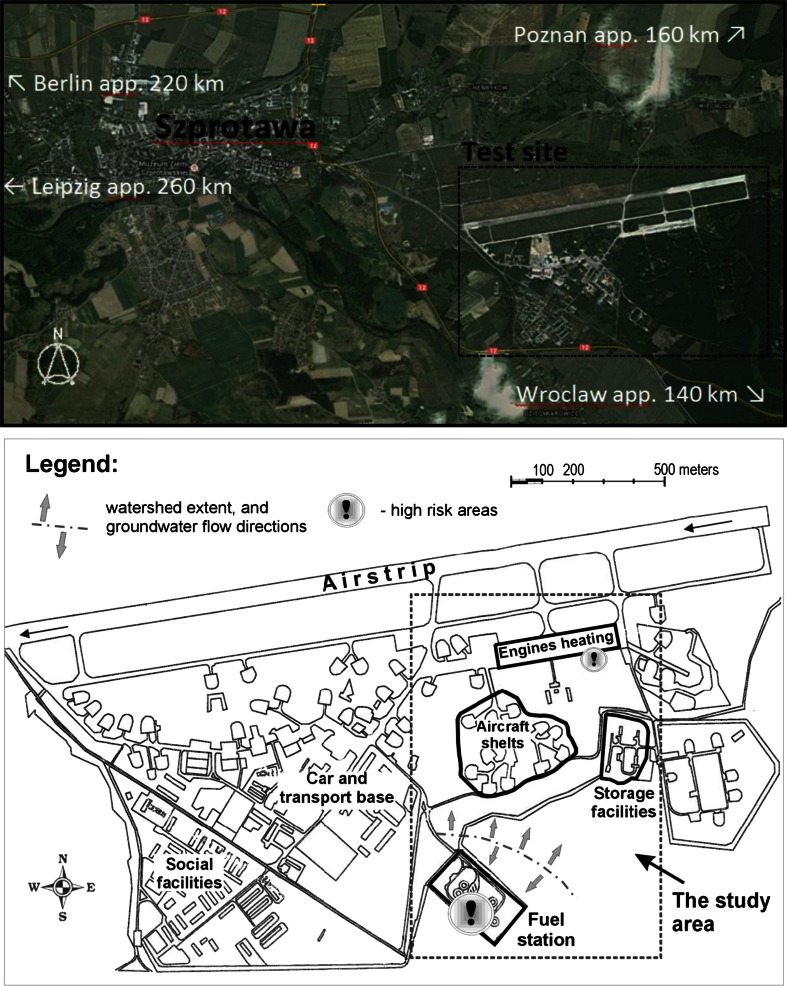
Location of the test site near Szprotawa (*top*) and the enlarged study area with zones of interest (*bottom*).

The study was performed on a former military airbase located in Szprotawa in southwestern Poland (Fig. 1). The airfield was established in the 1930s—initially intended for gliders. By World War II, it changed into a German military airbase. After the war, the airfield became a Soviet military airbase, hosting also nuclear weapons. After the collapse of communism, the site was left derelict. Since 1992, some parcels of the site have been reused by civil facilities as a residential and industrial zone, but most of the site has remained abandoned and is now dilapidated.

The derelict areas were investigated in the 1990s, revealing pollution with fuel compounds near the storage and distribution facilities for jet fuel (refueling, underground fuel storage, and pipeline facilities) (NFOŚ 1991). In 1998 and 1999, a purification of fuel compounds was performed, as well as a partial remediation by installing a bioventing barrier in the southwestern part of the former fuel station (Arkadis 1998). Assumably, high-risk areas are located in the central part of the airbase; see Fig. 1. A small pilot study (7–8 July 2011) showed that a considerable degree of pollution by jet fuel compounds was still present in the study area. In the soil, concentrations of benzines (light hydrocarbon fraction) between 1000 and 9000 mg/kg d.m. and BTEX concentrations of 50 to 70 mg/kg d.m. were measured in the high-risk areas (fuel station, aircraft engine heating area). Outside of these spots, the concentrations were significantly lower (benzines 11–23 mg/kg dry matter (d.m.), BTEX 2–2.3 mg/kg d.m.) but still exceeded acceptable standards for soil quality (Polish Ministry of Environment 2002; Supplementary Table S1) according to the planned future land redevelopment (housing or commercial services in accordance with a local spatial development plan).

Lithologically, the study area is covered by relatively homogeneous quaternary deposits, consisting of a thin layer of silty cover sediments (1–2 m) that are underlain by a 5–10-m layer of sands and gravels (aquifer). The free groundwater table is present at depths of between 0.2 and 7 m, typically in the range 2-3 m (Clean Air 1996). Besides the zones paved with concrete, the area is a combination of wastelands and forested areas. The groundwater depths measured during the present study were between 1.5 and 2.2 m below ground level (bgl) in the fuel station area, in southeastern direction dipping to 3.5 m bgl. In north and northeastern direction from the watershed, the groundwater table depths were between 1.2 and 2.0 m. On the watershed, the groundwater depths were in the range of 0.9–1 m bgl. The hydraulic conductivities of the aquifer ranged between 1.1 × 10^−6^ and 2 × 10^−4^ m/s.

### Field sampling

Field sampling was performed in two campaigns. The first campaign was between 11 and 20 September 2012 and the second campaign on 18 and 19 September 2013. The daytime temperature was from a minimum of 10 °C to a maximum of 27 °C. The weather was dry in the first campaign and with occasional drizzles in the second campaign. For the multiple pre-screening approaches, tree core sampling, soil gas measurements, and the DP high-resolution technologies of MIP and LIF were applied in the study area. Additionally, 20 piezometers for continuous groundwater monitoring were installed in groundwater wells, and, to confirm possible contaminations, individual groundwater samples were collected during direct-push operations, as well as soil samples from selected sampling points. In total, 220 sampling points were investigated with one or more of the screening methods. At 24 points, four or more different methods were applied.

### Methods’ descriptions

#### Tree core sampling

This method had its beginnings decades ago (Vroblesky et al. 1999), and a number of guidelines were published by several authors within the last couple of years (Algreen and Trapp 2014; Trapp et al. 2012; Holm et al. 2011; Vroblesky 2008). Tree cores from 17 willows (*Salix* sp.) and 18 aspen (*Populus tremula*) were sampled. The samples were collected at a height of 1 m. They had a length of 6 cm and were taken with a 6-mm increment borer (Suunto, Finland). Replicate samples were collected from the other side of the tree. The outer centimeter of each core sample was removed to avoid atmospheric influence followed by quick transfer of the remaining wood sample into 20-ml analytical vials with 4 ml of water. Subsequently, 0.5 ml internal standard containing fluorobenzene was added. The vials were closed and cooled until chemical analysis approximately 4–6 days after the sampling. All samples were analyzed for BTEX by headspace gas chromatography/mass spectrometry (HS-GC/MS) on an Agilent system with a 30 m × 0.25 mm × 1.00 μm ZB-5 capillary column (Phenomenex). Incubation and temperature program are described in Algreen and Trapp (2014). The average of replicates was used, and measurements above the detection limit but below quantification limit were set to one half of the quantification limit. Values are given in micrograms per liter (content of the vial), which corresponds to about 0.133 μg/kg wood when wood density is 1 kg/l.

#### Soil gas measurements

Active soil gas measurements were conducted at 84 sampling points by drilling a temporary probe (ø 36 mm) to a depth of 2 m. To avoid contamination by exhaust fumes, an electric drill hammer was used. The drill holes were subsequently sealed at the surface with a pneumatic packer, and the soil gas was pumped for 10 min with an electric pump at a flow rate of 4 l/min. During the pumping, the concentrations of oxygen (O_2_), methane (CH_4_), carbon dioxide (CO_2_), and hydrogen sulfide (H_2_S) were monitored using a Fresenius airTOX gas measurement system. Throughout the pumping period, the total ionizable gas was measured using a MiniRAE 3000 photoionization detector (PID, calibrated with isobutylene). The PID readings were documented at intervals of 0.5, 1, 2, 3, 5, 7, and 10 min. At every sampling point, a free air measurement was conducted and ambient air temperature and atmospheric pressure were documented. At four selected measuring points, soil gas extraction tests were conducted. Temporary gas monitoring wells were drilled by percussion drilling to a depth of 2 m and equipped with 2-in PVC piezometers. The piezometers were installed in a way that at least 1 m of filter section was available in the sandy unsaturated zone between the top layer and the groundwater level. Gas was extracted using a vacuum blower that was connected to the extraction well. The extraction tests were carried out for 20 min during which under pressure (hPa), flow rates (m^3^/h), and gas constituents such as oxygen, methane, carbon dioxide, and hydrogen sulfide (Fresenius airTox gas measurement system) as well as ionizable gases (PID) were constantly monitored.

At relevant PID concentrations during the extraction tests, gas samples were transferred into suitable containers (vacuum bottles, sorbent tubes) and taken to a commercial laboratory (AGROLAB Labor GmbH, Bruckberg, Germany) for quantitative analysis of BTEX, benzine range hydrocarbons (C_5_–C_12_), and chlorinated hydrocarbons by gas chromatography. Samples in the sorbent tubes were extracted with phenoxyethane/methanol; samples in the vacuum bottles were measured directly. The extracts obtained were analyzed by GC/MS using a Varian 3900 system with a Varian Saturn 2100T mass spectrometer and a CP-SIL5CB 25 m × 0.15 mm × 2.0 μm df capillary column. The resulting mass spectra were confirmed with the NIST database using a search software and considering the calculated match probability. Each analysis was validated with a blank sample. Signals appearing in both the sample and the method blank were not considered. The temperature program was as follows: 40 °C ramped at 15 °C per min to 200 °C held for 13 min. The injection volume was 1-ml headspace volume.

#### Direct-push with high-resolution sensors

Using DP, soil, soil gas, and groundwater samples were taken, and various sensors (for example, MIP, Geoprobe, and LIF, Dakota Technologies) were added to the DP equipment. MIP was used for in situ screening of chlorinated hydrocarbons (CHCc) and other VOCs in both the saturated and the vadose zones. Separation and detection of compounds is by gas chromatography equipped with a PID, flame ionization detector (FID), and a dry electrolytic conductivity detector (DELCD). This detector combination allowed for a selective specification of the contaminant type. LIF is able to detect every contamination caused by oil-derived hydrocarbons. The sensors were drilled down to 10 m bgl using a Geoprobe DT6620 drill rig with optional anchored bridge for the hydraulic hammer, and 14 MIP and 26 LIF profiles were taken down to a maximum of 10 m bgl. Before each sounding, a testing of the system sensitivity was carried out (MIP and LIF specific). Cone penetration testing (CPT) was applied, too, to determine soil properties and to map the site-specific lithology.

#### Soil and groundwater sampling and monitoring

Soil and groundwater sampling included the collection of 7 soil samples and 19 groundwater samples with direct-push and the installation of 20 groundwater monitoring wells. Soil samples were collected using a hand rig (70-mm diameter). The samples were taken from the vadose zone and/or the groundwater level zone. To avoid evaporation of volatile compounds, the samples were directly placed in sealed glass vessels of 300-ml volume for analyses on hydrocarbons. At five different investigation points, soil sampling was performed in a depth-differentiated manner with four samples per location (Geoprobe DT22 sampling system). Two types of HDPE piezometers were installed for groundwater sampling and monitoring. All groundwater monitoring wells (15 2″ ID and 5 1″ ID microwells) were installed down to around 7 m bgl with 4–5-m screened intervals and 60-μm filter protection membrane to prevent clogging. Bentonite seals were placed above the screens up to the surface. Thirty-nine depth-differentiated groundwater samples (Geoprobe SP16 sampling system) were pumped up. Soil and groundwater samples were directly placed in sealed dark glass vessels (300 ml) to minimize evaporation and degradation of the compounds. These samples were shipped to the lab in a refrigerator at +4 °C. VOCs were extracted from soil (1 g) with methanol (5 ml) in closed vials by agitation. After sedimentation, an aliquot of the extract (10–250 μl) was injected to 9.75 ml of deionized water in a chromatographic vial. The final volume was adjusted to 10 ml by adding methanol. BTEX and gasoline were determined by head space-solid phase microextraction (HS-SPME) on a polydimethylsiloxane (PDMS) fiber on a Shimadzu system with a 60 m × 0.25 mm DB-5MS capillary column. To determine the amount of VOCs in the water, an aliquot of the sample was placed in a chromatographic vial (the aliquot volume depended on VOC concentration), and the final volume was adjusted with deionized water to 9.75 ml. Methanol (250 μl) was added to improve the dissolution of VOCs. The next steps of the analytical procedure were the same as in the determination of VOCs in soil. Field measurements of temperature, dissolved oxygen, electrical conductivity, and pH were done with an EC Professional Plus 1700/1725 from YSI (USA), and of oxidation reduction potential with either ORP200 from HM Digital (USA) or SenTix ORP electrode from WTW GmbH (Germany). The results obtained by soil and groundwater sampling were compared to those from the other methods.

#### Data treatment and comparison of methods

Results were mapped using the software program Surfer 10. Contour plots were created by interpolation with the Kriging gridding method. The relationship between the results from the various methods was quantified by the nonparametric Spearman rank correlation. Only results obtained at nearby sampling point (located within 15 m or less) were compared. The concentrations measured in different tree species that had been sampled at close-by sampling points were compared by a two-tailed *t* test with an error probability of 0.05 (α = 5 %).

## Results

### Individual applications

#### Tree core sampling

**Fig. 2:**
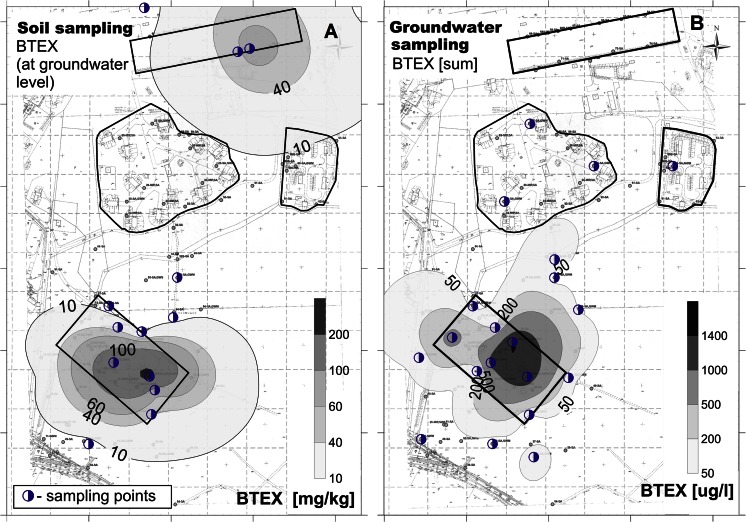

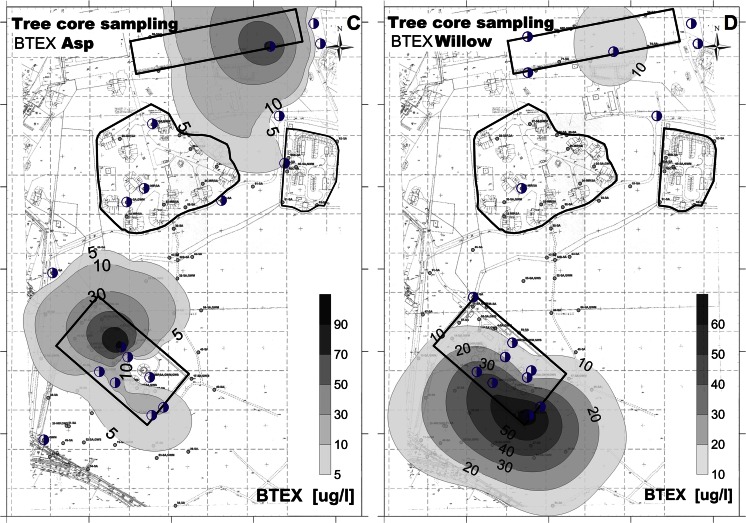

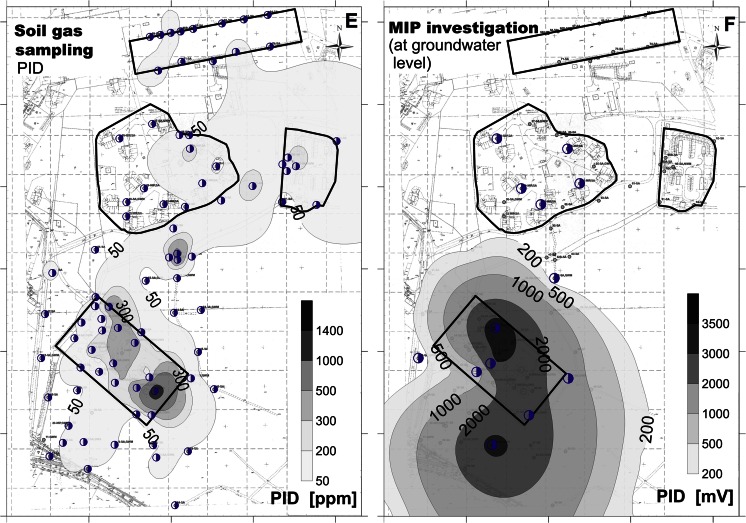
Maps of the BTEX contamination at Szprotawa obtained by different sampling methods: **a** soil sampling, **b** groundwater monitoring, **c** tree coring by aspen, **d** tree coring by willows, **e** soil gas measurements, and **c** MIP/LIF. *Dots* refer to sampling sites

BTEX were measurable in both willow and aspen, though in relative low concentrations. The concentration intervals were as follows: benzene < quantification limit (QL) (0.40 μg/l) to 31.67 μg/l; toluene < QL (0.079 μg/l) to 86.43 μg/l; ethylbenzene ≤ QL (0.079 μg/l) to 37.36 μg/l; m,p-xylene < QL (0.16 μg/l) to 47.02 μg/l; and o-xylene < QL (0.39 μg/l) to 18.82 μg/l. Toluene was detected in all samples, ethylbenzene and xylenes were detected in most of the samples (26 and 24 out of 35 samples, respectively), and benzene in some of the samples (8 out of 35 samples). The highest concentrations were measured in samples taken at or next to the former fuel station and near the east end of the former aircraft engine heating area; see Fig. 2c, d. Samples from both tree species were collected and compared at 10 sampling points. BTEX were taken up more by willows than by aspen, and the difference in uptake was significant for toluene and xylenes (*t* test, α = 5 %) (Supplementary Table S2).

Growth inhibition of trees was observed around the former fuel tanks, a site with high subsurface pollution: Few trees had remained, and those were small and miserable, stunned, and some were withered. Subsurface gasoline can lead to growth inhibition (Trapp et al. 2001).

#### Soil gas measurements

Concentrations of gases measured as by-products of the natural degradation process in the former fuel station area were determined in the range of 1.6–6.6 % CH_4_ (Supplementary Fig. S2a), 2.5–15.5 % CO_2_ (Supplementary Fig. S2b), and 0.2–2.6 ppm H_2_S. Oxygen concentrations were in the range of 6.1–16.8 %, i.e., below the O_2_ concentration of 21 % in the ambient air. The concentrations of ionizable gases (PID) in the highly polluted areas were between 166 and 1630 ppm, showing stable readings during the 10 min of pumping (Fig. 2e). The remaining area was characterized by measured gas concentrations in the range of 0.4–6.2 % (CO_2_), 0–0.7 ppm (H_2_S), and 11.5–20.3 % (O_2_). Methane was found in four profiles with CH_4_ concentrations of 0.1–0.6 % (Supplementary Fig. S2a). The PID index was measured in a range of 1.6–220 ppm, with a trend of concentrations decreasing during the 10-min interval. The interpolation plot in Fig. 2e shows the results obtained by the soil gas PID measurements.

The chemical composition of the soil gas at selected sampling points is summarized in Supplementary Table S3. The data show a generally good correlation between the on-site PID measurements and the concentrations of VOCs (mainly jet fuel hydrocarbons, C_5_–C_12_) measured in the gas phase. The investigated soil gas samples are characterized by generally low concentrations of BTEX compounds, although slightly elevated concentrations of benzene (0.4 mg/m^3^), toluene (0.4 mg/m^3^), ethylbenzene (1.0 mg/m^3^), and xylene (1.5 mg/m^3^) were evident in samples from the former fuel area. These samples also showed high to very high concentrations of jet fuel hydrocarbons (C_5_–C_12_) between 310 and 2100 mg/m^3^. The sample from outside the fuel station was characterized by slightly elevated CO_2_ concentrations (1.45 %), but generally low concentrations of other VOCs. Chlorinated hydrocarbons were not detected in any of the soil gas samples.

#### Direct-push and direct sensing methods

Inside the area of the former gas station, MIP signals showed enhanced tailing effects, probably caused by the light non-aqueous phase liquid (LNAPL) of kerosine swimming on the top of the groundwater. Outside the former fuel station, the MIP profiles displayed a normal behavior with a significantly increased ability in terms of vertical resolution capacity. LIF proved to be the best choice for delineating free phase total petroleum hydrocarbons (TPHs, here benzine or kerosine). The LIF technology delivered sharp signals and depth reliable detections and hence proved to be an effective method for obtaining a source zone inventory. Therefore, LIF results were used for the 3D evaluation of the spatial extension of the benzine body in the area of the former fuel station. Two-dimensional vertical cross sections, horizontal distributions of contamination, lithology, and hydraulics were generated. The 3D architecture of the contaminant body based on different LIF threshold values is displayed in Supplementary Fig. S1. LIF profiles could be calibrated and validated by lab analytics on total petroleum content in soil. LIF total fluorescence proved to match the petroleum content of soil samples taken in the area of the former fuel station. Based on the developed relationship, the whole petroleum body still present at the site within the former refueling area was estimated to contain about 200 t of petroleum within a volume of approximately 80,000 m^3^.

#### Soil and groundwater sampling

Analyses of groundwater and soil samples allowed a comparison to the findings from other methods and a quantification of the contaminants in the study area. Benzines were found in highest concentrations around and at the former fuel station. Benzine concentrations were up to 11,145 mg/kg in soil samples, with median at 1160 mg/kg, and thus provided the bulk of pollution. Benzines in groundwater (GW) monitoring wells were found in 24.1 mg/l, median at 5.16 mg/l. In GW samples, benzines were up to 93 mg/l, median at 7.24 mg/l. The highest concentrations of BTEX in soil samples were confirmed for the area of the former fuel station, and at GW level. The concentrations ranged between 70 and 240 mg BTEX/kg. Elevated levels of BTEX were also measured near the former aircraft engine heating area. An interpolation plot based on the results obtained by soil sampling 1 m bgl is shown in Fig. 2a. Figure 2b shows the results obtained by groundwater measurements. The sum of BTEX in the monitoring wells at the former fuel station ranged between 1200 and 2200 μg/l. Outside the highly polluted areas, BTEX concentrations were measured in a range of 0.1–95 μg/l.

Measurements of the redox potential displayed deeply anaerobic conditions of the groundwater in the fuel station hot spot area (redox potential range of −120 mV, in the remaining area up to +100 mV), convergent with the lowest dissolved oxygen (DO) readings (4–6 % of saturation, in the remaining areas up to 30 % DO) and elevated temperatures (12.0–14.5 °C, compared to the usual 11.0 °C). This effect was also noticeable in the southern and southwestern part of the aircraft shelters’ area; however, its intensity was lower.

#### Summary

The combination and comparison of the results derived with the various methods generated knowledge on the site’s contamination status. The results are highly reliable and low effort was needed to create the information. Notably, it was confirmed by all methods that the area of the former fuel station is a highly polluted area. Figure 2 presents the resulting spatial patterns of pollution derived by the individual approaches. The first impression is that all methods—besides being more or less costly to apply in terms of time and materials needed—generate a comparable 2D pattern of the pollution level. The delineation of the polluted area depends of course on the location of the sampling points. Hence, the hot spot near the engine heating area in the right upper corner was not detected by all methods.

### Statistical comparison of characterization methods

Rank correlation analysis was applied to question or to confirm the impression of similarity between the results obtained by the different methods. Rank correlation was chosen, because it can test the monotone trend also of non-equally distributed data, and of data with different units. Both tree coring and soil gas measurements were used for rapid initial detection of contaminants in the subsurface. A comparison of BTEX in tree cores with the concentrations of gases in soil (CH_4_, CO_2_, O_2_, H_2_S, and PID measurement) collected at the same sampling points indicates high rank correlations between the two methods (Table 1). The correlation is negative for O_2_. The correlations were significant at α = 5 % with one exception (tree coring aspen with PID).

**Table 1:** Rank correlation between the sum of BTEX measured in tree core samples of willows and aspen and soil gas measurements; *n* = 8

Soil gas measurement	Tree core sampling	
	Willow	Aspen
CH_4_ (%)	**0.734**	**0.609**
CO_2_ (%)	**0.857**	**0.889**
O_2_ (%)	**−0.905**	**−0.760**
H_2_S (ppm)	**0.734**	**0.916**
PID after 5 min of pumping (ppm)	**0.889**	0.320

Bold indicates significant correlation at α = 5 %

*PID* photoionization detector

Table 2 presents the Spearman rank correlation coefficients between analysis results for sum of BTEX in samples from groundwater monitoring wells or groundwater samples (taken with direct-push), and in tree cores and in soil gas (by MIP and LIF). All correlations between BTEX in groundwater monitoring samples with that in tree cores, and with those from soil gas PID and MIP, were significant at α = 5 %. The correlations to BTEX obtained by groundwater sampling were positive, but below the significance level. The correlation between the sum of BTEX in soil samples and in tree cores was positive and significant at α = 5 %, while no significant correlation was found for PID in soil gas measurements. The rank correlation coefficients for the individual compounds in tree cores are reported in Supplementary Table S4. Correlations to concentrations in tree cores of individual compounds are higher for results from groundwater monitoring than for results from groundwater sampling with DP.

**Table 2:** Rank correlation between groundwater monitoring, groundwater and soil sampling, and screening methods for sum of BTEX

	Tree core sampling	Soil gas measurement(PID after 5 min of pumping)	MIP (max PID)	LIF (max fluorescence)
Groundwater monitoring	*n* = 6	*n* = 18	*n* = 9	*n* = 2
BTEX - sum	***0.7714***	**0.6987**	**0.8333**	Too few data
Groundwater sampling	*n* = 5	*n* = 17	*n* = 2	*n* = 7
BTEX - sum	0.5000	0.2219	Too few data	0.000
Soil sampling	*n* = 14	*n* = 6		
BTEX - sum	***0.6748***	−0.200		

Bold means significant rank correlation at α = 5 %. Bold and italic mean significant rank correlation at α = 10 %

*PID* photoionization detector, *MIP* membrane interface probe, *LIF* laser-induced fluorescence, *BTEX* benzene, toluene, ethylbenzene, and xylene

## Discussion

The pre-screening methods applied in this study are cost-efficient semi-quantitative site characterization approaches. Additionally, both tree core and soil gas sampling are fast and low-invasive techniques. Tree coring allows two persons to obtain 50 samples per day in case of optimum site conditions. Both tree core and soil gas sampling have been successfully applied for the discovery and delineation of subsurface benzine and BTEX spills. In general, data from tree coring and soil gas surveys can be used to gain and increase knowledge of the nature and extent of contamination at a site, as well as to guide the placement of high-resolution sensors or other, far more expensive quantitative measures such as groundwater wells.

### Statistical comparison

A normal distribution is unusual for concentration data derived from contaminated sites: Consistently, high concentrations are found inside the polluted areas, whereas outside concentrations will typically be very low, thus creating skewed (non-normal) distributions of data (Wahyudi et al. 2012). Indeed, for most data used in this study, the mean is higher or even far higher than the median, confirming skewed distributions (Supplementary Table S5). Methods using rank statistics have been suggested to overcome this problem (Wahyudi et al. 2012). Hence, rank correlation is to be preferred over Pearson correlation (Hauke and Kossowski 2011). Our results indicate that a significant rank correlation between the various site inspection methods is not always found for the Szprotawa data set. Possible reasons may include the semi-quantitative character of some of the methods or the fact that the methods are based on different sampling techniques. However, the most likely reason is assumed to be related to the different sampling depths and locations. We also included results from zero samples into the correlation analysis, i.e., samples in which no compound was detected in the sample even though the compound could have been measured by the quantitative methods, which substantially affected the statistical outcome in a negative way. A low and insignificant cross-covariance and lacking spatial correlation between tree core sampling and groundwater monitoring were observed by Wahyudi et al. (2012) at another site. The reason for such a low correlation is not only due to the high variability of the results obtained from the tree core samples. Groundwater monitoring typically has a small sample grid and sample volume and thus may fail to yield positive contaminant findings, where trees, due to the large underlying root zone volume and the high sample density, indicate pollution. At that site, the tree core analysis was capable to indicate the plume distribution, allowed to detect hot spots (Larsen et al. 2008), and was even leading to the discovery of a new, beforehand undetected plume (Wittlingerova et al. 2013). The advantage of a high sampling size that can be obtained with little effort also holds true for soil gas measurements and, although to a lesser degree, also for the direct-push methods.

### Identification of hot spot areas

Even though a significant rank correlation was not always found between the different investigation methods, the interpolation plots show (Fig. 2a–f) that all pre-screening methods allowed the identification of the most polluted area, which is the former fuel station. Even the measurement of methane CH_4_ and/or oxygen O_2_ in soil gas already indicated areas with high subsurface activity and thus an increased likelihood of pollution (Supplementary Fig. S2a, b).

### A multiple pre-screening approach compared to the conventional approach

A conventional screening approach based upon (available) historical data helps to gain information about selected areas. Groundwater sampling/monitoring and/or soil sampling with a limited budget leads to a wide sampling grid resulting in sparse data. This entails an enhanced risk of overlooking single hot spots or even high-risk areas, which in consequence can make decision-making risky and difficult (Wycisk et al. 2013; Rein et al. 2011). In contrast, the application of multiple pre-screening steps results in more data which will be more targeted as well. This minimizes the risk of missing single hot spots/high-risk areas without increasing the financial burden. In this study, results obtained by tree coring indicated a second area of high contamination levels: the aircraft engine heating area. The contamination in that area was then confirmed by additional soil sampling. This area would have remained undetected in a conventional screening approach based on historical data as no such data pointed to this area. No groundwater and soil samples would have been taken due to the lack of a historical indication and given limited budgets for a complete dense field investigation. This emphasizes the advantage of applying at least one of the inexpensive pre-screening methods that are able to cover wide areas with reasonable efforts.

**Fig. 3:**
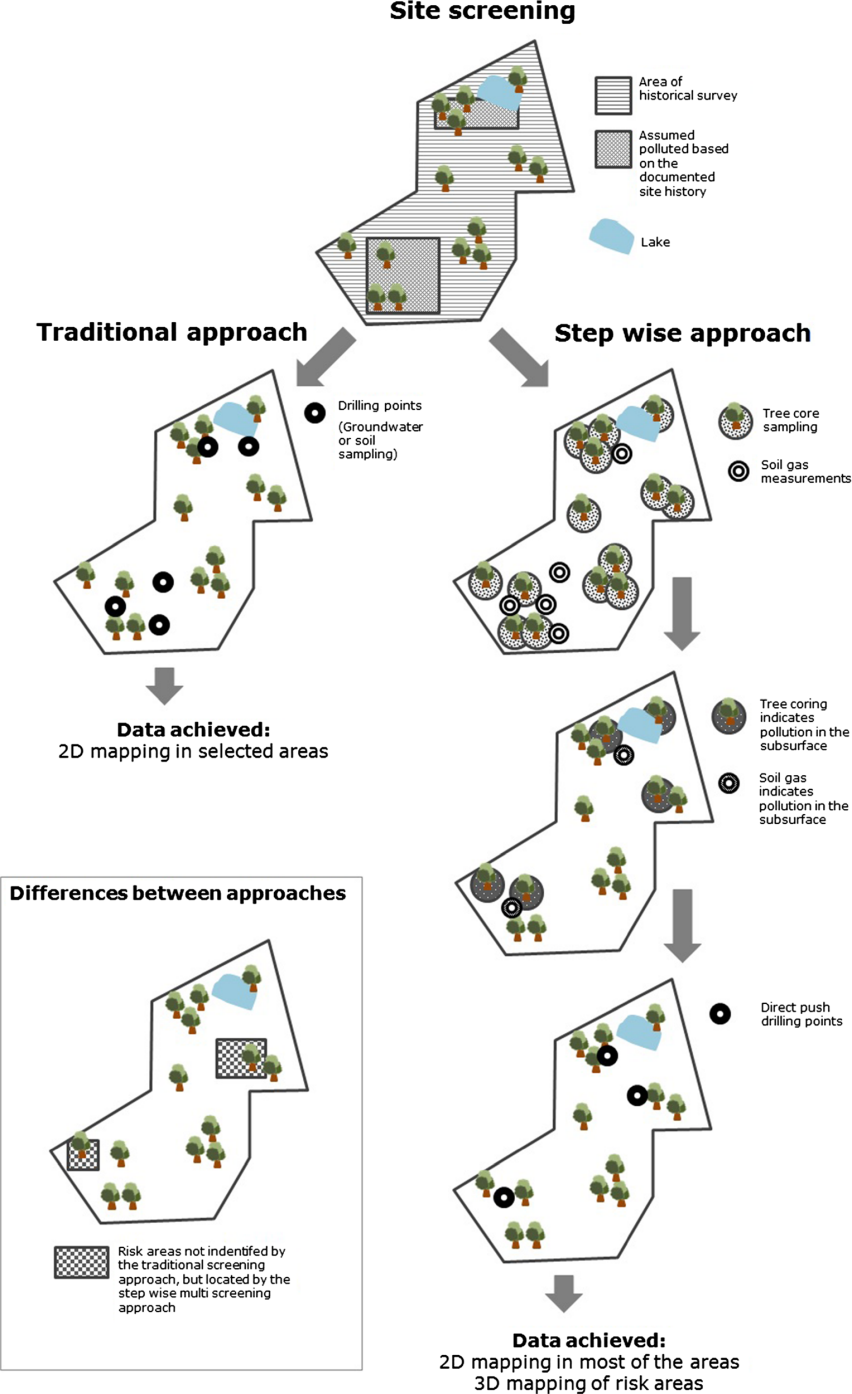
Combination of site investigation approaches. *Top left*: traditional screening; *top right*: stepwise multi-screening; *bottom*: indication of potential risk area overseen by the traditional screening approach but located by the stepwise multi-screening approach

We therefore advise that an efficient strategic approach to a successful site characterization should start with methods that imply the lowest (application) costs before using more precise and expensive methods in the pre-identified (highly) polluted areas or areas of highest concern. Tree core sampling and soil gas measuring are rapid and inexpensive methods, which can be applied as initial pre-screening methods all over an entire study area as long as the conditions at the test site allow their application. Based on the results obtained by these two pre-screening methods, MIP and/or LIF technologies can then be applied in selected areas for which vertical data are of interest or needed. Soil and/or groundwater sampling with chemical analysis should be applied as the last step to confirm an identified contamination and to quantify the contaminant levels. Figure 3 illustrates the conventional screening approach opposed to an approach applying stepwise multiple pre-screening methods.

### Site characterization and remediation strategy

Site characterization is not only a matter of localization and quantification of a suspected pollution. Site characterization can also provide valuable data for the design of further remediation approaches and thus save efforts and money (Kästner et al. 2012). From tree coring, the feasibility of phytoremediation at the actual site can be estimated by upscaling the processes and fluxes with mathematical models (Trapp et al. 2014). Soil gas measurements of methane and carbon dioxide also provide quick information concerning ongoing natural degradation processes. In the absence of oxygen, organic pollutants in the subsurface are converted to methane by microorganisms through the process of methanogenesis. Through the influx of atmospheric oxygen, methane is subsequently degraded to carbon dioxide (e.g., Rettenberger 1995). At the Szprotawa site, this process has resulted in a typical zonation pattern: Methane is concentrated in the hot spot areas and in the depth, and it is surrounded by a halo of increased carbon dioxide concentration. Soil gas measurements are only feasible when sufficient gas can be extracted from soil. The applicability of this screening method is directly related to the feasibility of the air sparging remediation method. With this method, also known as in situ air stripping, air is injected into the subsurface to extract hydrocarbons via the vapor phase. The direct-push sensors (e.g., MIP and LIF) give in situ vertical information about the contamination levels, but also about the hydraulic and geological properties of the underground (Leven-Pfister et al. in Kästner et al. 2012). Based on the findings of the screening methods reported in this paper, in a next step, remediation scenarios were evaluated for the Szprotawa site, which will be reported in Clausen et al. (2015).

### Limitations

The step-wise multiple screening approach shown in Fig. 3 cannot always be applied in full scale, due to properties of the site. Limitations are the absence of trees (tree coring impossible), high groundwater levels (application of soil gas measurements limited), rocky soil (application of direct-push sensors limited), or hazardous subsurface due to former land use, e.g., explosives as in the case of Szprotawa (direct-push methods limited). Another limited field of application for tree core sampling and soil gas measurements is pollutions located in great depths, i.e., beyond the range of tree roots and too deep to affect the shallow soil gas zone (typical for dense non-aqueous phase liquids (DNAPLs) such as chlorinated solvents). However, the depth range of phytoscreening goes beyond the root zone. Chlorinated solvents from groundwater in a depth of 12.5–19 m bgl were already detected in the tree cores (Sorek et al. 2008). Own observations (unpublished, obtained during the study of Larsen et al. (2008)) include trichloroethylene (TCE) signals in wood from TCE spills in groundwater more than 30 m bgl, even though the average maximum rooting depth of coniferous and deciduous trees in temperate zones is only 3.9 and 2.9 m (Canadell et al. 1996).

## Conclusions

We applied and compared a sequence of site characterization methods for a former Soviet military airbase, where fuel contamination was expected to be found in shallow groundwater and subsoil. The methods applied and compared included phytoscreening by tree coring; soil gas measurements for CO_2_, CH_4_, O_2_, and PID; the direct-push sensors MIP and LIF; direct-push sampling; and sampling from soil and from groundwater monitoring. The rapid and inexpensive pre-screening methods of phytoscreening and soil gas measurements both indicated subsurface pollution and hot spots and are considered successful in this case. Also, BTEX were found by applying these methods. Trichloroethylene (TCE) and methyl tert.-butyl ether (MTBE), which were also analyzed in wood, were not detected and were not present in other samples either (true negatives). The application of direct-push sensors (MIP and LIF) yielded 3D information about the extension and the volume of the subsurface plume. The LIF sensor calibrated for kerosine gave sharp signals for the delineation of the free phase TPH (here, kerosine). Groundwater levels, hydraulic characteristics, and soil texture properties were also determined with direct-push. The comparison of the results from conventional soil and groundwater sampling (from wells) with those of the chemical analysis confirmed the results of the pre-screening and direct-push methods: At the site, jet fuel (benzines, light alkanes) and BTEX were present in groundwater and soil (2 m bgl), partly in high concentrations. In this study, we expanded the applicability of tree coring to BTEX compounds and tested the use of high-resolution direct-push sensors. In this way, we gained experience that increases the trust in the applicability and the reliability of these new methods for megasite investigation.

Besides site characterization, the goal of the study was to optimize the interplay between new rapid screening methods and conventional site characterization methods. It can be confirmed that a large-scale application of non- or low-invasive pre-screening can help in directing and focusing the subsequent, more precise but also more expensive methods. The application of rapid pre-screening also led to the identification of an unexpected, beforehand undetected polluted area (the engine heating station) far away from the expected hot spot area (the fuel station). Such areas may remain undetected without the application of an extensive, large-scale pre-screening (due to time and budget constraints), leading to a suboptimal and risky decision about site management. Moreover, the rapid pre-screening methods also yielded useful information about potential remediation methods. Tree coring can indicate the efficiency of phytoremediation (concerning both phytoextraction and root zone remediation). Soil gas measurements show ongoing natural attenuation (via formation of methane and other by-products) and indicate the feasibility of air sparging and bioventing. Direct-push methods yield additional information relevant for remediation, such as groundwater level, volume of contamination, and subsurface properties.

## Electronic supplementary material

ESM 1 (DOC 4327 kb)
