# Supplementary material for: Using pre-screening methods for an effective and reliable site characterization at megasites
Source: Environ Sci Pollut Res Int. 2015 May 17;22(19):14673–86. doi: 10.1007/s11356-015-4649-6 (PMC4592496; doi:10.1007/s11356-015-4649-6)
Supplement: Supplementary file 1 — (DOC 4327 kb) [file 11356_2015_4649_MOESM1_ESM.doc]

**Supplementary material**

**Using pre-screening methods for an effective and reliable site characterization at megasites**

**Mette Algreen^a,+^, Mariusz Kalisz^b,+^, Marcel Stalder ^c^, Eugeniu Martac^d^, Janusz Krupanek^b^, Stefan Trapp^a,*^, Stephan Bartke^e^.**

a Department of Environmental Engineering, Technical University of Denmark, 2800 Kgs. Lyngby, Denmark

b Institute for Ecology of Industrial Areas, 6 Kossutha Street, Katowice, Poland

c SolGeo AG, Dornacherplatz 3, 4501 Solothurn, Switzerland

d Fugro Consult GmbH, Volkmaroder Str. 8c, 38104 Braunschweig, Germany

e Helmholtz Centre for Environmental Research – UFZ, Permoserstr. 15, 04318 Leipzig, Germany

+ both authors contributed equally to this work

**Table of Contents**

Table S1. Standards for soil quality defined in the Polish law for specific land use functions

Table S2. Species-differences in uptake of BTEX by willow and aspen

Table S3. Laboratory data of soil gas at selected sampling points

Table S4. Rank correlation between screening measurements for BTEX and single compounds

Table S5. Maximum, mean and median of all results obtained

Figure S1. Spatial distribution of NAPL within the area of the former fuel station derived from LIF sensing

Figure S2a. Result of soil gas measurements for methane CH_4_

Figure S2b. Result of soil gas measurements for carbon dioxide CO_2_

**Table S1.** Standards for soil quality defined in the Polish law for specific land use functions

| **Type of land use** | **Depth** | **BTEX** | **Benzines (C_6_-C_12_)** |
| --- | --- | --- | --- |
|  | [m bgl] | mg/kg d.m. | |
| **C group**  Industrial use/ production related services | 0-2 | **200** | **500** |
|  | 2-15 | **10** | **50** |
| **B group**  agricultural and forestry, urbanized/ housing areas, commercial services | 0-0.3 | **0.1** | **1** |
|  | 0.3-15 | **1** | **5** |
| **A group**  areas subjected to protection under the regulations of Nature Conservation | no specified | **0.1** | **1** |

**Table S2.** Species-differences in uptake of BTEX by willow and aspen.

|  | Benzene | | Toluene | | Ethylbenzene | | o-xylene | | m,p-xylene | |
| --- | --- | --- | --- | --- | --- | --- | --- | --- | --- | --- |
| Sample ID | Asp | Willow | Asp | Willow | Asp | Willow | Asp | Willow | Asp | Willow |
| 2.2 | 0.00 | 0.00 | 1.65 | 17.2 | 0.00 | 0.32 | 0.00 | 0.64 | 0.32 | 0.31 |
| 2 | 0.00 | 0.00 | 1.02 | 13.0 | 0.00 | 0.00 | 0.20 | 0.00 | 0.08 | 0.00 |
| 4 | 0.00 | 17.4 | 8.96 | 26.4 | 0.04 | 7.21 | 0.18 | 0.98 | 0.28 | 13.7 |
| 5 | 0.00 | 0.00 | 1.45 | 1.07 | 0.09 | 0.00 | 0.00 | 0.00 | 0.00 | 0.00 |
| 11 | 0.00 | 0.00 | 0.00 | 0.08 | 0.00 | 0.00 | 0.00 | 0.00 | 0.00 | 0.00 |
| 12 | 0.00 | 0.00 | 0.18 | 0.06 | 0.02 | 0.00 | 0.00 | 0.00 | 0.08 | 0.00 |
| 13 | 0.00 | 0.00 | 0.66 | 2.90 | 0.01 | 0.01 | 0.00 | 0.20 | 0.08 | 0.00 |
| 17 | 0.00 | 0.47 | 2.93 | 14.7 | 0.77 | 3.99 | 0.52 | 3.58 | 0.85 | 15.4 |
| 18 | 0.67 | 10.5 | 11.2 | 37.1 | 0.08 | 0.29 | 0.34 | 1.31 | 0.43 | 1.34 |
| 19 | 0.00 | 1.46 | 11.8 | 26.0 | 0.32 | 4.28 | 0.25 | 4.61 | 0.40 | 11.9 |
| F-test | 0.00 | | 0.01 | | 0.00 | | 0.00 | | 0.00 | |
| T-test | 0.16 | | 0.04 | | 0.11 | | 0.08 | | 0.04 | |
| Significant difference  among the species | No | | Yes | | No | | Yes | | Yes | |
| Rank correlation coefficient | 0.471 | | 0.903 | | 0.487 | | 0.781 | | 0.751 | |
| Significant (5%) | No | | Yes | | No | | Yes | | Yes | |

**Table S3.** Laboratory data of soil gas at selected sampling points.

| **Compound** | **Sampling Point** | | |  |
| --- | --- | --- | --- | --- |
|  | **P2** | **P21** | **P85** |  |
| CH_4_ [%] | 2.8 | 0.0 | 0.7 | Field values |
| CO_2_ [%] | 5.5 | 1.45 | 15.5 |  |
| O_2_ [%] | 12.3 | 19.7 | 5.1 |  |
| PID [ppm] | 365 | 29.7 | 1355 |  |
| Sum chlorinated HC [mg/m^3^] | n.d. | n.d. | n.d. | Lab. values |
| Hydrocarbons C5-C12 [mg/m^3^] | 310 | <5.0 | 2100 |  |
| Benzene [mg/m^3^] | <0.2 | 0.2 | 0.4 |  |
| Toluene [mg/m^3^] | 0.4 | <0.2 | <0.2 |  |
| Ethylbenzene [mg/m^3^] | 1.0 | <0.1 | <0.1 |  |
| Xylene [mg/m^3^] | 1.5 | <0.2 | 0.4 |  |

**Table S4.** Rank correlation between screening measurements for BTEX and single compounds. Bold = Significant rank correlation at = 5 %. Bold and Italic Significant rank correlation at α = 10%.

|  | **Tree core sampling** |  |  |  |
| --- | --- | --- | --- | --- |
| **Groundwater**  **monitoring [**ug/L**]** | n=6 |  |  |  |
| BTEX - sum | ***0.7714*** |  |  |  |
| Benzene | 0.4472 |  |  |  |
| Toluene | 0.3714 |  |  |  |
| Ethyl benzene | ***0.7201*** |  |  |  |
| Xylene | **0.8452** |  |  |  |
| **Groundwater**  **sampling [**μg/L**]** | n=5 |  |  |  |
| BTEX - sum | 0.5000 |  |  |  |
| Benzene | -0.1768 |  |  |  |
| Toluene | 0.5000 |  |  |  |
| Ethyl benzene | 0.1000 |  |  |  |
| Xylene | 0.4000 |  |  |  |
| **Soil sampling [mg/kg dw]** | n=14 |  |  |  |
| BTEX - sum | **0.6748** |  |  |  |

**Table S5.** Maximum, mean and median of all results obtained.

| **Soil gas analysis** | max | mean | median |
| --- | --- | --- | --- |
| Temp. [°C] | 27.0 | 19.2 | 20.0 |
| CH4 [%] | 6.60 | 0.30 | 0.00 |
| CO2 [%] | 15.50 | 2.44 | 1.45 |
| O2 [%] | 20.70 | 17.82 | 19.50 |
| H2S [ppm] | 2.80 | 0.25 | 0.10 |
| Free air measurement | 5.70 | 0.78 | 0.40 |
| PID after 30'' of pumping [ppm] | 1455 | 136 | 74 |
| PID after 1' of pumping [ppm] | 1569 | 116 | 54 |
| PID after 2' of pumping [ppm] | 1632 | 101 | 30 |
| PID after 3' of pumping [ppm] | 1601 | 94 | 23 |
| PID after 5' of pumping [ppm] | 1513 | 87 | 18 |
| PID after 7' of pumping [ppm] | 1429 | 82 | 14 |
| PID after 10' of pumping [ppm] | 1355 | 79 | 13 |
| SA Date | 41171 | 41137 | 41165 |
| SA Time | 0.81 | 0.58 | 0.59 |
| Depth [m] | 2.00 | 1.97 | 2.00 |
| GW-Level [m] | 1.90 | 1.83 | 1.90 |

| **GW monitoring** | max | mean | median |
| --- | --- | --- | --- |
| BTEX - sum [ug/l] (nov-2011) | 2201 | 639 | 532 |
| benzene [ug/l] (nov-2011) | 28.3 | 4.9 | 1.2 |
| toluene [ug/l] (nov-2011) | 48.6 | 21.6 | 23.5 |
| ethylbenzene [ug/l] (nov-2011) | 549 | 141 | 76 |
| xylene [ug/l] (nov-2011) | 1614 | 471 | 437 |
| BTEX - sum [ug/l] (may-2012) | 1347 | 483 | 312 |
| benzene [ug/l] (may-2012) | 18.1 | 3.5 | 0.6 |
| toluene [ug/l] (may-2012) | 80.2 | 23.2 | 6.5 |
| etylobenzene [ug/l] (may-2012) | 448 | 118 | 44 |
| xylene [ug/l] (may-2012) | 1021 | 338 | 238 |
| benzines (may-2012) | 24086 | 9614 | 5155 |
| BTEX - sum [ug/l] (sept-2012) | 1423 | 267 | 30 |
| benzene [ug/l] (sept-2012) | 19.1 | 3.5 | 0.5 |
| toluene [ug/l] (sept-2012) | 22.9 | 9.5 | 9.8 |
| etylobenzene [ug/l] (sept-2012) | 506 | 85 | 17 |
| xylene [ug/l] (sept-2012) | 907 | 187 | 18 |
| benzines [ug/l] (sept-2012) | 17812 | 7054 | 7136 |

| **GW sampling** | max | mean | median |
| --- | --- | --- | --- |
| BTEX - sum [ug/l]  (GWS nov2011_1.9-2.9 m) | 4711 | 2733 | 2143 |
| benzene [ug/l] (GWS nov2011_1.9-2.9 m) | 57 | 34 | 43 |
| toluene [ug/l] (GWS nov2011_1.9-2.9 m) | 99 | 24 | 11 |
| etylbenzene [ug/l] (GWS nov2011_1.9-2.9 m) | 1450 | 818 | 734 |
| xylene [ug/l] (GWS nov2011_1.9-2.9 m) | 3211 | 1856 | 1383 |
| BTEX - sum [ug/l] (GWS nov2011_5-6 m) | 1826 | 1173 | 1179 |
| benzene [ug/l] (GWS nov2011_5-6 m) | 294 | 125 | 46 |
| toluene [ug/l] (GWS nov2011_5-6 m) | 12.3 | 6.8 | 7.6 |
| etylbenzene [ug/l] (GWS nov2011_5-6 m) | 562 | 410 | 538 |
| xylene [ug/l] (GWS nov2011_5-6 m) | 959 | 650 | 614 |
| BTEX - sum [ug/l] (GWS sept2012) | 4794 | 1009 | 159 |
| benzene [ug/l] (GWS sept2012) | 74 | 31 | 10 |
| toluene [ug/l] (GWS sept2012) | 224 | 33 | 19 |
| etylbenzene [ug/l] (GWS sept2012) | 1626 | 293 | 28 |
| xylene [ug/l] (GWS sept2012) | 3089 | 652 | 120 |
| benzines [ug/l] (GWS sept2012) | 93010 | 20499 | 7235 |

| **Soil sampling** (mg/kg dm) | max | mean | median |
| --- | --- | --- | --- |
| Soil (GW level) benzene | 1.70 | 0.26 | 0.06 |
| Soil (GW level) toluene | 10.5 | 1.91 | 0.82 |
| Soil (GW level) ethylbenzene | 79 | 14 | 3 |
| Soil (GW level) xylene | 160 | 39 | 34 |
| Soil (GW level) sum BTEX | 240 | 55 | 41 |
| Soil (GW level) benzines | 11145 | 2310 | 1160 |
| Soil Benzines | 1.05 | 1.05 | 1.05 |
| Soil sum BTEX in 1 m depth | 4.32 | 2.27 | 2.27 |
| Soil benzines in 1 m depth | 115.00 | 11.87 | 6.19 |

| **µg per tree core** | max | mean | median |
| --- | --- | --- | --- |
| Benzene | 31.7 | 4.7 | 0.7 |
| Toluene | 86.4 | 9.5 | 2.7 |
| Ethylbenzene | 37.4 | 2.7 | 0.3 |
| m,p-xylene | 47.0 | 5.1 | 0.7 |
| TCS o-xylene | 18.8 | 2.3 | 0.7 |
| Naphthalene | 2.1 | 0.8 | 0.4 |
| MTBE | 0.0 | 0.0 | 0.0 |
| TCE | 0.0 | 0.0 | 0.0 |

|  |  | max | mean | median |
| --- | --- | --- | --- | --- |
| **MIP** | MIP depths (m bgl) | 5.79 | 2.72 | 2.70 |
| **MIP** | MIP max (PID in mV) | 3588 | 1331 | 878 |
| **LIF** | UVOST depths (m bgl) | 2.82 | 2.33 | 2.25 |
| **LIF** | UVOST max (Fluorescence in %) | 147 | 64 | 70 |


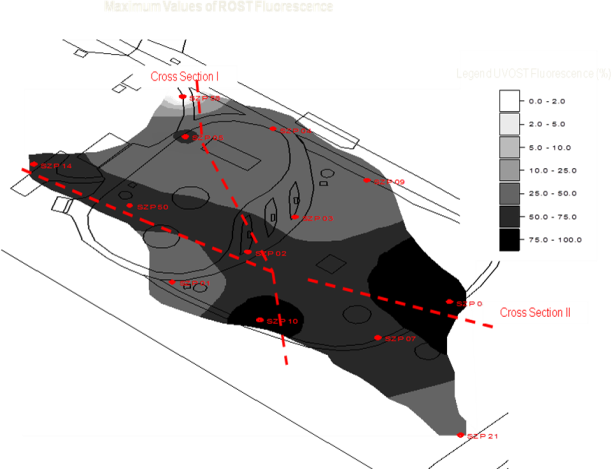

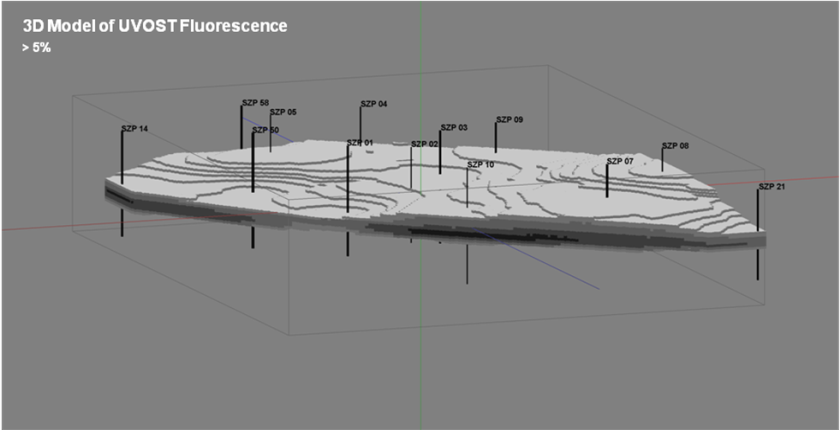


**Figure S1.** Spatial distribution of NAPL within the area of the former fuel station derived from LIF sensing.


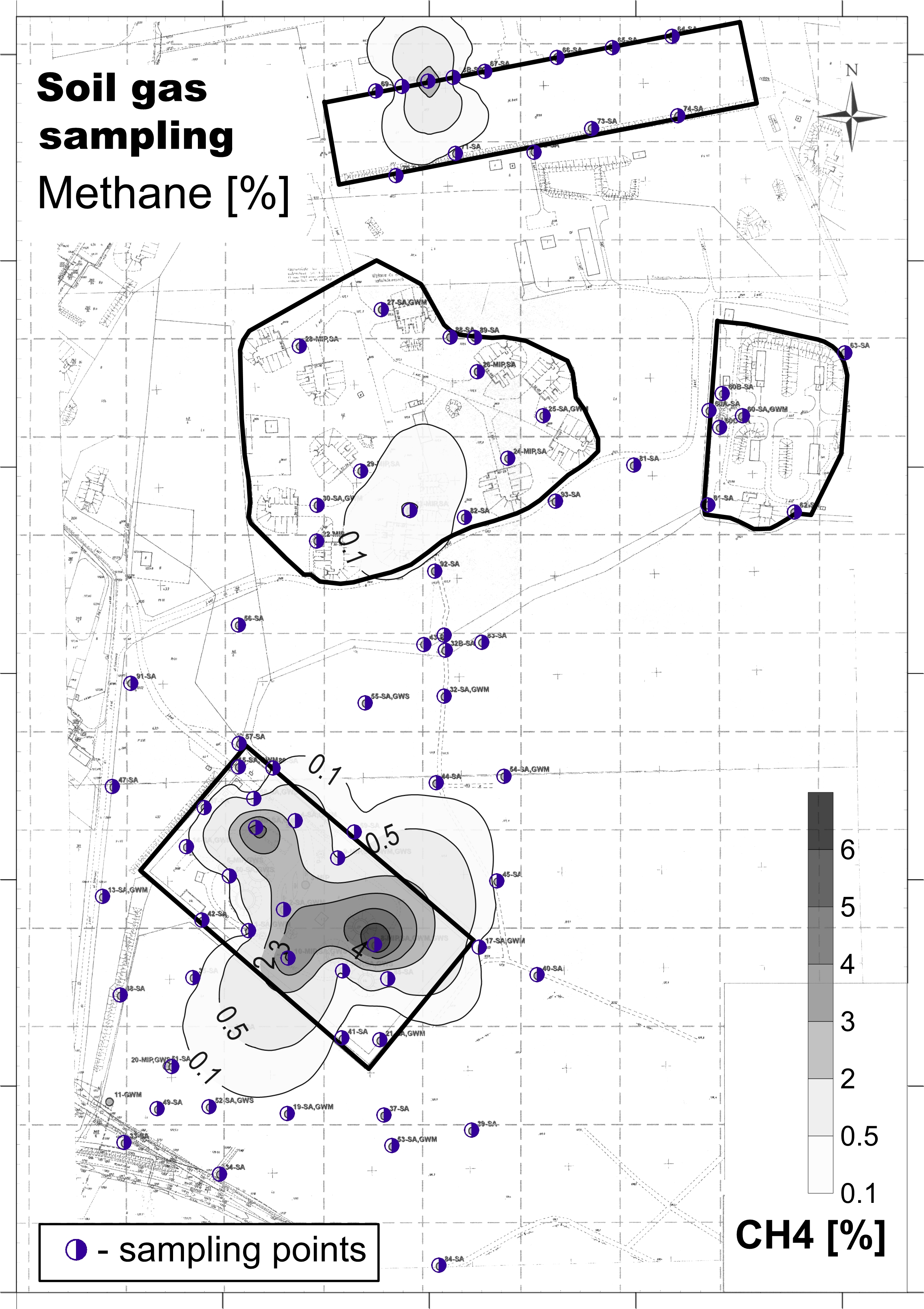


**Figure S2a.** Result of soil gas measurements for methane CH_4_.


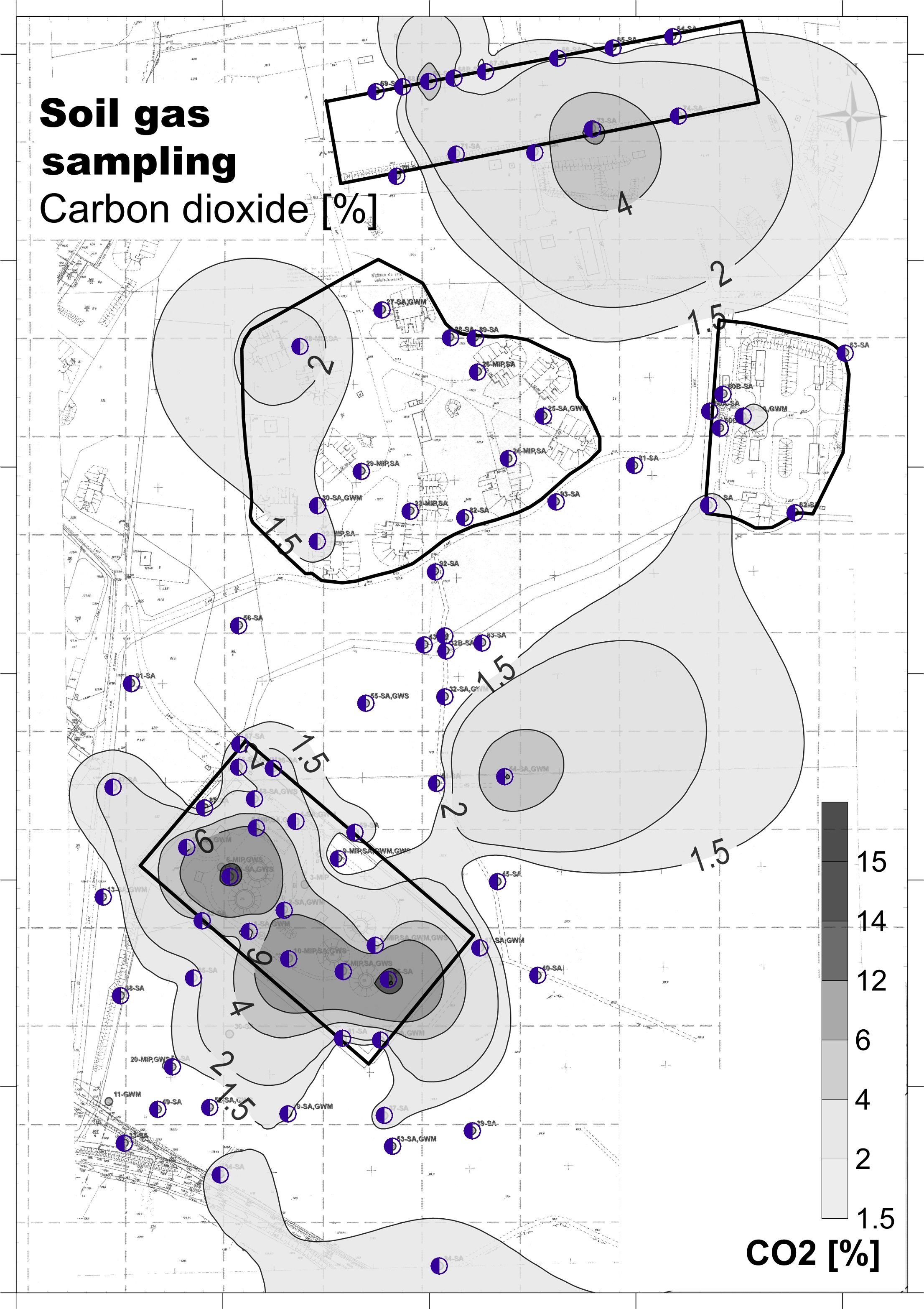


**Figure S2b.** Result of soil gas measurements for carbon dioxide CO_2_.
